# Geological carbon storage site characterization using a dual element seismic recording technology

**DOI:** 10.1038/s41598-025-96012-8

**Published:** 2025-04-15

**Authors:** Alireza Malehmir, Magdalena Markovic, Tanni Juul Abramovitz, Ulrik Gregersen

**Affiliations:** 1 Department of Earth Sciences, Uppsala University, Uppsala, Sweden; 2 Geological Survey of Denmark and Greenland, Copenhagen, Denmark

**Keywords:** Seismology, Geophysics

## Abstract

Geologic carbon storage in saline acquirers is a feasible and scalable way of reducing atmospheric carbon dioxide (CO_2_). Since 2022, Denmark has stepped up site characterization at five suitable onshore locations for this purpose with a particular focus on four-way domal structures. A dual-element recording system incorporating landstreamer and wireless recorders was innovated and upscaled in a cost- and time-effective way for this purpose at the Rødby structure (one of the five sites). The dual-element recording allows a better imaging of the near-surface structures but also because of its broadband nature, it helps to retain higher resolution for imaging toplap structures and smaller faults in the area. The landstreamer data better image fault structures offsetting the Bunter Sandstone Formation, which is the primary reservoir, and the overlying geological seals such as Fjerritslev, Ørslev and Falster Formations. The Vedsted Formation, which a secondary seal in the region, appears unfaulted in the landstreamer data. The landstreamer data also reveal glacial valleys, near the surface, which are a source of groundwater. The two complementary datasets, from the landstreamer and nodal data, help to de-risk geological carbon storage in Denmark and is a solution we recommend to be adapted for onshore sites elsewhere in the world specially where the logistical acquisition challenges are not significant.

## Introduction

**Fig. 1:**
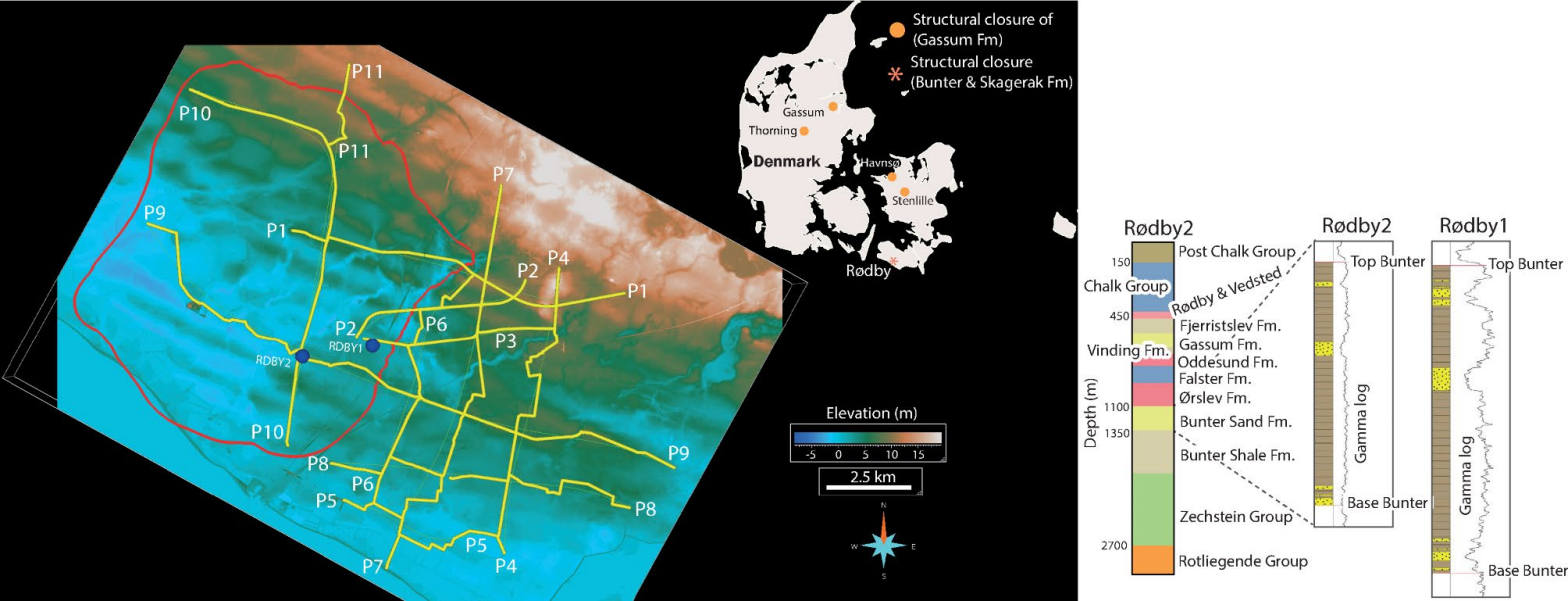
LiDAR (light detection and ranging) elevation map showing the acquired seismic profiles (P1-P11) in the Rødby region (southern Denmark) and an earlier interpreted outline^11^ of the domal structure (red polygon) used for the planning of new seismic profiles. The outset figure shows the expected stratigraphy (e.g., the target Bunter Sandstone Formation) observed from Rødby 1 and 2 wells. Important for this study are Ørslev, Falster, Fjerritslev and the very thin layers of Rødby and Vedsted, which are primary, secondary, tertiary and quaternary seal formations, respectively. The Lower Cretaceous Rødby and Vedsted formations are very thin, and their base forms an uncomfortable surface above the Lower Jurassic Fjerritslev Formation. Seismic data along P10 and P11 (jointly processed along a N-S strike) are primarily presented in this work. Illustrator and SKUA-GOCAD software are used to prepare the figure. LiDAR data were provided by the Geological Survey of Denmark and Greenland (GEUS). Generic Mapping Tools (GMT) V4.5 (https://www.soest.hawaii.edu/gmt/) was partly used to produce the figure.

Carbon capture and storage (CCS) is a fundamental solution for climate change mitigation^1–3^ and geological carbon storage (GCS) is a feasible and scalable way of reducing atmospheric carbon dioxide (CO_2_) to meet the Paris climate action agreement^4^. European Union aims for 250 million tons of CO_2_ to be stored annually within the Economic Area by 2040, requiring safe and permanent storage with no risk to health and environment^5^. Among the binding countries, Denmark has made an ambitious and accelerated effort to reach the Paris agreement 1.5°C objective. According to the Danish Energy Agency and the government, Denmark aims to reduce 70% of its emission by 2030 and reaches a climate neutrality by 2050, providing quality baseline data, and attracting international investments^6–8^. Thanks to its geology, Denmark hosts numerous sedimentary four-way domal structures formed as a result of salt tectonics mainly during and partly after the Mesozoic^9,10^ (Fig. 1). These salt dome structures greatly control later sedimentary depositions. A series of sandstone reservoirs with porosities up to 20–30% such as Gassum and Bunter Sandstone formations were formed prior to the main structural doming due to salt pillow growth. These saline reservoirs can allow between 12–22 billion tons of CO_2_ to be stored in the Danish subsurface, optimistically compensating for 400–700 years of Denmark’s CO_2_ emissions^11,12^. However, the salt withdrawal and up-doming has resulted in local extensional settings with sometimes multiple grabens and normal faults originating from the salt core and often showing several minor splay faults. These faults, if unsealed, are a potential risk for CO_2_ leakage.

For CO_2_ to be at a supercritical phase, target reservoirs are usually at depth greater than 800–850 m depth to hold a pressure around 7–8 MPa^13–17^. In Denmark, the Gassum Formation (Upper Triassic-Lower Jurassic occurring at 1000–1500 m depth) is the most favorable formation for GCS applications such as in Stenlille, Havnsø, Gassum and Thorning^11^ (Fig. 1). At these sites, the Fjerritslev Formation (thick mudstone overlying the Gassum Fm) is the primary seal formation, and Rødby and Vedsted formations, and lower parts of late Cretaceous carbonates of the Chalk Group, are combined a secondary seal. The overlying Chalk Group (Upper Cretaceous-Lower Paleocene) has over 800 m thickness. In contrary, at the Rødby structure, the Bunter Sandstone Formation (Lower Triassic) is the primary saline reservoir and lies approximately 500 m below the Gassum formation. The Chalk Group has much less thickness (approximately 300 m) because it has been eroded away over 400 m thick due to uplift and halokinesis and only a portion of Triassic and Jurassic sediments are present today^18^. The Rødby-1 and -2 wells drilled near the crest of the Rødby dome show some undifferentiated Upper Cretaceous rocks of Rødby and Vedsted formations (Fig. 1). At this location, erosion and hiatus is the highest rate, hence the Lower Jurassic Fjerritslev Formation and the Lower Cretaceous successions (Vedsted and Rødby formations) are the thinnest. Delineating these formations in high-resolution and if any fault penetrates through them is important for CCS development at the site. Gassum Formation is not favorable for CCS at the Rødby structure since it appears too shallow, around 650–700 m depth. The Bunter Sandstone Formation at about 1000–1200 m depth is more suitable for GCS. In this context, Fjerritslev Formation is a tertiary seal for CO_2_ migration at Rødby, and Ørslev and Falster formations with a similar thickness are a primary and secondary seals for the Bunter Sandstone Formation, respectively (Fig. 1). For simplification and in this article, we use only primary and secondary seal terms for the Bunter Sandstone Formation and when required we refer to the seal with its own formation name.

Seismic methods, due to good resolution at depth, are extensively used for site characterization, baseline and repeat surveys to characterize reservoirs, reservoir capacity and porosity estimation, imaging fault systems and for monitoring CO_2_ plumes^19–22^. Seismic methods are used in combination with reservoir engineering and modeling solutions to provide reliable capacity estimation. Today, offshore seismic surveys benefit from long and multiple seismic streamers for 3D surveying (usually 6–9 of them and as long as 5–8 km) and in some cases combined with ocean bottom seismometers (OBS) and ocean bottom cables (OBC) to allow improved imaging of fault systems from the target reservoir all the way to the seafloor^23,24^. The most promising and cost-effective technology to allow both near seafloor and deep imaging is to employ multi marine short- and long-streamers using hydrophones as the recording sensors with broad bandwidth exceeding 1000–2000 Hz. Hydrophone spacing for long marine streamers is between 10–50 m. Digital recording is not an issue for marine surveys as there is no cultural contaminations from power lines and infrastructures. Short marine streamers (100–750 m long depending on the sea water column) have smaller hydrophone spacing, from 2 to 12.5 m, and are simultaneously towed with the longer streamers to provide a full-depth range imaging capability for CCS applications^25,26^. On land, the situation is different, nodal recorders have made a tremendous step for improved imaging, however, deploying shortly spaced recorders are nor practical neither gives a good public impression, considered to be invasive. Fiber-optic cable through distributed acoustic sensing technologies (DAS) can be used for this purpose^27,28^ but at the characterization stage and as a surface array has limiting capabilities. DAS technology is still at its infancy stage for surface arrays and for regional site characterization, therefore, there has not been so much innovation and step forward for land GSC seismic applications. The push for more GSC site selections and CCS work implies a wide range of possibilities for countries with surface access and suitable geology.

To make onshore GCS characterizations more time and cost effective, in 2022 and adapting from a series of studies for fault imaging in mega-cities^29,30^, a pilot survey was conducted in the Stenlille-Havnsø domal structures to test up-scaling capability of a dual-element, nodal and landstreamer recording system for CCS applications in Denmark^31–33^. The dual-element recording has the advantage of providing short offset data (in this study a maximum of 215 m was used), unaliased, for first breaks to help better estimate refraction static solution and also mapping near-surface structures compared to conventional larger spacing receivers used for land data acquisition. Successful results let to a series of surveys totaling over 650 km/line high-resolution and high-fold data acquisition across five major CCS sites (Stenlille, Havnsø, Gassum, Rødby and Thorning; CCS2022-2024 projects of GEUS) onshore Denmark. While encouraging results have been showcased and used for interpreting the structures of these sites^34–36^, the dual-element setup used for GCS applications has not yet come to a full realization by others elsewhere. It has not been possible to fully convince the CCS developers of the added-value proposition of the seismic landstreamer used in conjunction with nodal recorders to resemble what the marine seismic technology would employ for full depth imaging possibilities. Thanks to the Rødby data and its geological setting, in this study, we can provide convincing and complementary results this dual-element recording system provides for GCS applications. This is possible because the Chalk Group (a high-velocity cover) is less thick, and hence it allows deeper penetration of seismic energy and improve imaging for the landstreamer data. We know this from two wells (Rødby wells 1 and 2; Fig. 1) where core samples have provided stratigraphic information of the area. At the Rødby site (Fig. 1), the landstreamer data (40–200 m long array was used) image in high-resolution, thanks to the broadband, digital recording, and finer sampling (2 m versus 10 m) spacing, down to the top Zechstein at 1200–1300 ms, which is about 1500–1700 m depth. This allows to demonstrate the contribution of the dual-element recording system for CCS applications on land, which is showcased in this paper.

## The dual-element land seismic data acquisition technology

**Fig. 2:**
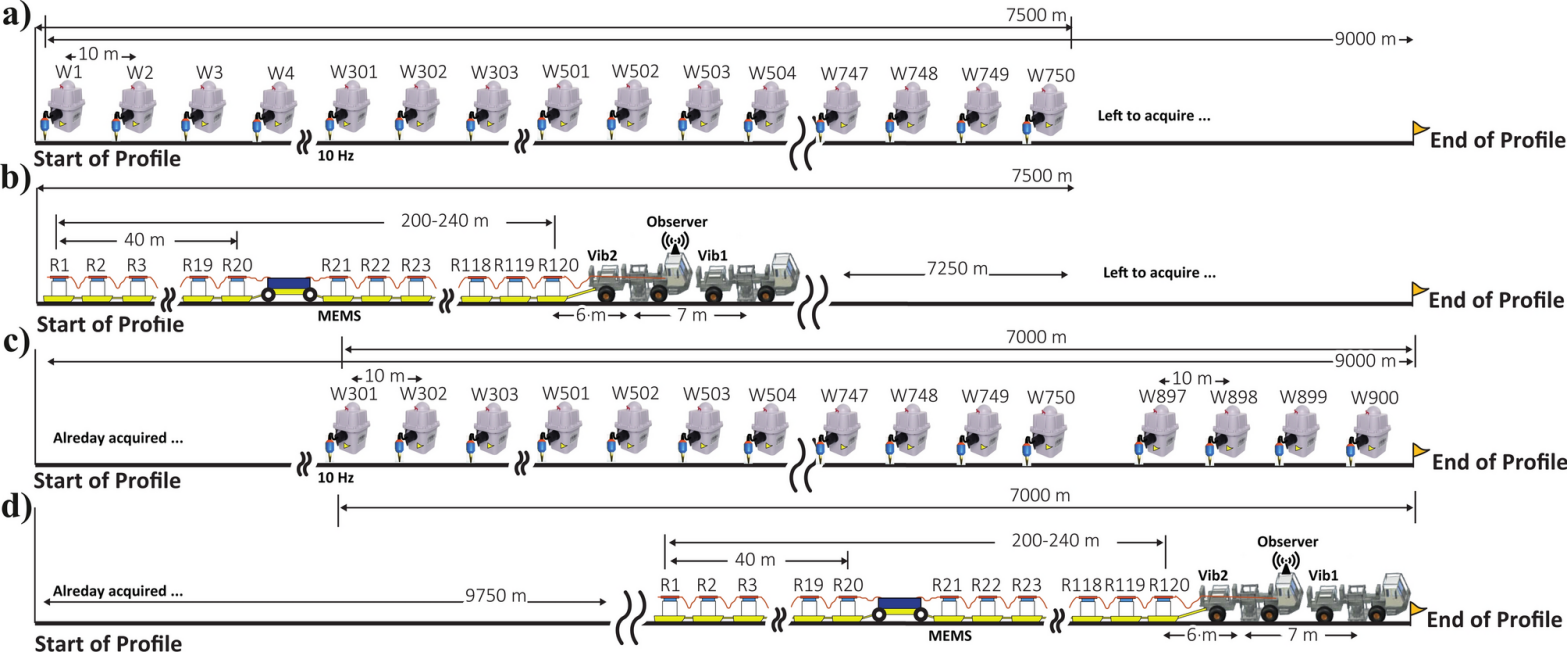
Sketch showing the data acquisition procedure of the dual-element recording system exemplified for a roll-along geometry. For a 9 km long profile, 750 nodal (wireless, labelled as W1, W2, …) recorders connected to 10-Hz geophone are first deployed. The landstreamer recorders (labeled as R1, R2, …) equipped with GPS antenna for time tagging and recording, towed by the rear vibro truck, which generate shots at every nodal recorder, 10 m spacing, and it continues for 250–300 shot points until in a predefined plan the first 300 nodal recorders are harvested for their data, charged and deployed in front of the array. The procedure continues until the profile is completely surveyed. Illustrator CC 2017 software (https://www.adobe.com/products/illustrator.html) was used to prepare the figure for this study.

The dual-element seismic acquisition setup comprises (1) a 2m-spaced MEMs (micro-electromechanical system)-based landstreamer, and (2) an often 10m-spaced nodal array (a remote autonomous unit, RAU, connected to geophones). Figure 2 shows a sketch of the data acquisition setup for a fixed nodal geometry i.e., the profile is not over 10 km and managing nodal arrays with 800–1000 modes feasible and practical.

**Fig. 3:**
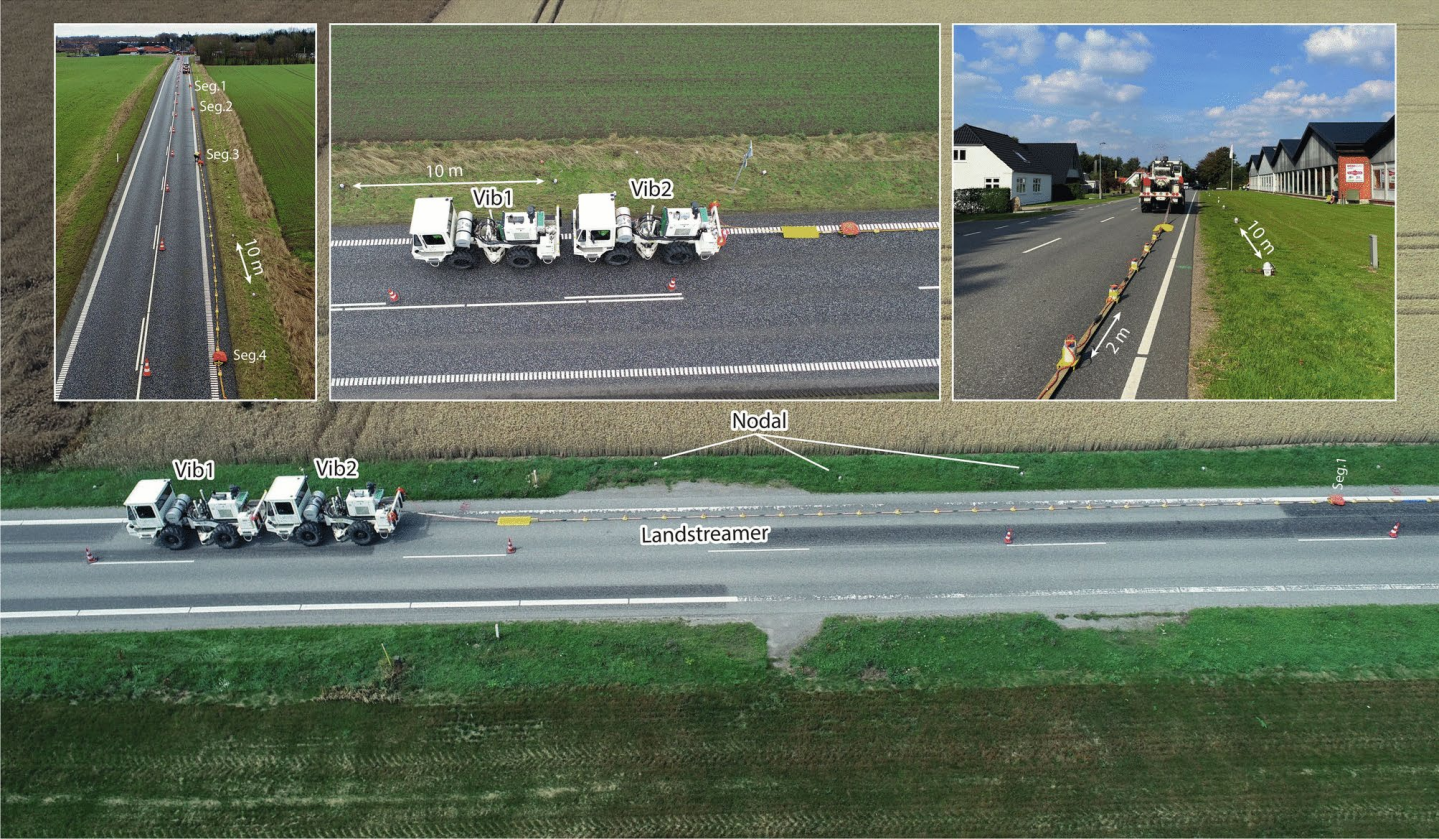
A series of field photos showing different parts of the dual-element acquisition setup including 4–5 segments (each 20 units and 2 m spacing) MEMs-based and nodal recorders (spaced at every 10 m) where shots were generated using two 12t mini-vibrators. Photos are taken by Alireza Malehmir.

Figure 3 shows a series of field photos from different elements of the recording system. The setup resembles what the marine seismic industry innovated as the short- and long-streamer layout for improved shallow and deeper imaging applications in deep-sea waters. To ensure penetrating the Chalk Group and providing good imaging capabilities down to the Bunter formation, at the Rødby site, and based on our earlier experience from the pilot studies, to two 12t mini vibrators (Vib1 and Vib2) were used to generate the seismic signal with Vib2 towing the landstreamer and an observer performing data quality and triggering the data acquisition. The landstreamer is equipped with GPS (global positioning system) antenna for accurate GPS-time tagging and sampling. The GPS-time tagging of the streamer data is used for harvesting identical data from the nodal recorders operating autonomously along the active part of the seismic profile. In Rødby, depending on the length of the profiles (Fig. 1), a fixed (e.g., if < 8 km) or an asymmetric split-spread roll-along geometry for the nodal array was used. Nominally, 700–750 nodal recorders were used live for any shot record providing a maximum CDP (common midpoint) fold of 350. As for the landstreamer, depending on the complexity of the road and logistics, up to 100 units were used providing a maximum CDP fold of 50.

Within 40 days, over 110 km of dual-element data were acquired using three repeated shots (to improve signal-to-noise ratio) generated at every shot point (10 m spacing, at every nodal position) with 18 s linear sweeps ranging 10–140 Hz. The cross-correlated data (with the pilot sweep), reduced to 5 s and vertically stacked for the repeated shot records resulted in over 8 million traces for the nodal and 600 thousand for the landstreamer recorders, respectively. This is an industry-scale data recording outcome and encouraging given the period it took to acquire the data. The question remains if the landstreamer data add any special value for GCS purposes, which we address now. Table 1 shows the key acquisition parameters used in the Rødby survey.

**Table 1:** Key elements of the acquisition parameters of the Rødby seismic survey, June–July 2023.

Survey parameters		
Recording system	Sercel Lite	
Source	INOVA UNIVIB-326 (2 × 12t, 95 kN per truck)	
Source sweep	10 Hz to 140 Hz linear sweep over 18 s, 3 sweeps per shot point	
Shot point spacing	10 m	
Geodetic surveying	Reach RX RTK DGPS	

Recording parameters	Landstreamer recorders	Nodal recorders
Receiver spacing	2 m	10 m
Spread type	End-on spread	Fixed to asymmetric split-spread
Offset (near, far) (average)	(15 m, 215 m)	(0 m, 7500 m)
Receiver	MEMS-3C	10 Hz spike (RAU-1C)
Sampling interval	1 ms	2 ms
Record length (before cross-correlation)	25 s	25 s
Record lenght (after cross-correlation)	5 s	5 s

## Nodal versus landstreamer raw data

There are fundamental differences between the two recording elements. The landstreamer recorders are MEMS-based (hence digital) and broadband (0–400 Hz) while the nodal recorders are connected to 10-Hz geophones (analogue, bandlimited and sensitive to electrical noises). There is a scope here to replace the geophone-based nodal recorders with MEMs-based or broadband nodal recorders, however, they were unavailable to us when the data were acquired. Future surveys can benefit from this additional recording element. Other than that, (1) the streamer recorders are often placed on roads (i.e., harder surfaces) than the nodal recorders (often planted into soils), and (2) the streamer recorders have shorter spacing, 2 m as opposed to the nodal recorders having 10 m spacing; the short spacing is important to ensure full bandwidth recording of the seismic wavefield and unaliased data^37,38^. The source and input signals are otherwise identical. To illustrate the contribution of the landstreamer data, we first compare example raw shot gathers, from P10, then with brute stacks, since minimal processing is applied for both recording elements.

**Fig. 4:**
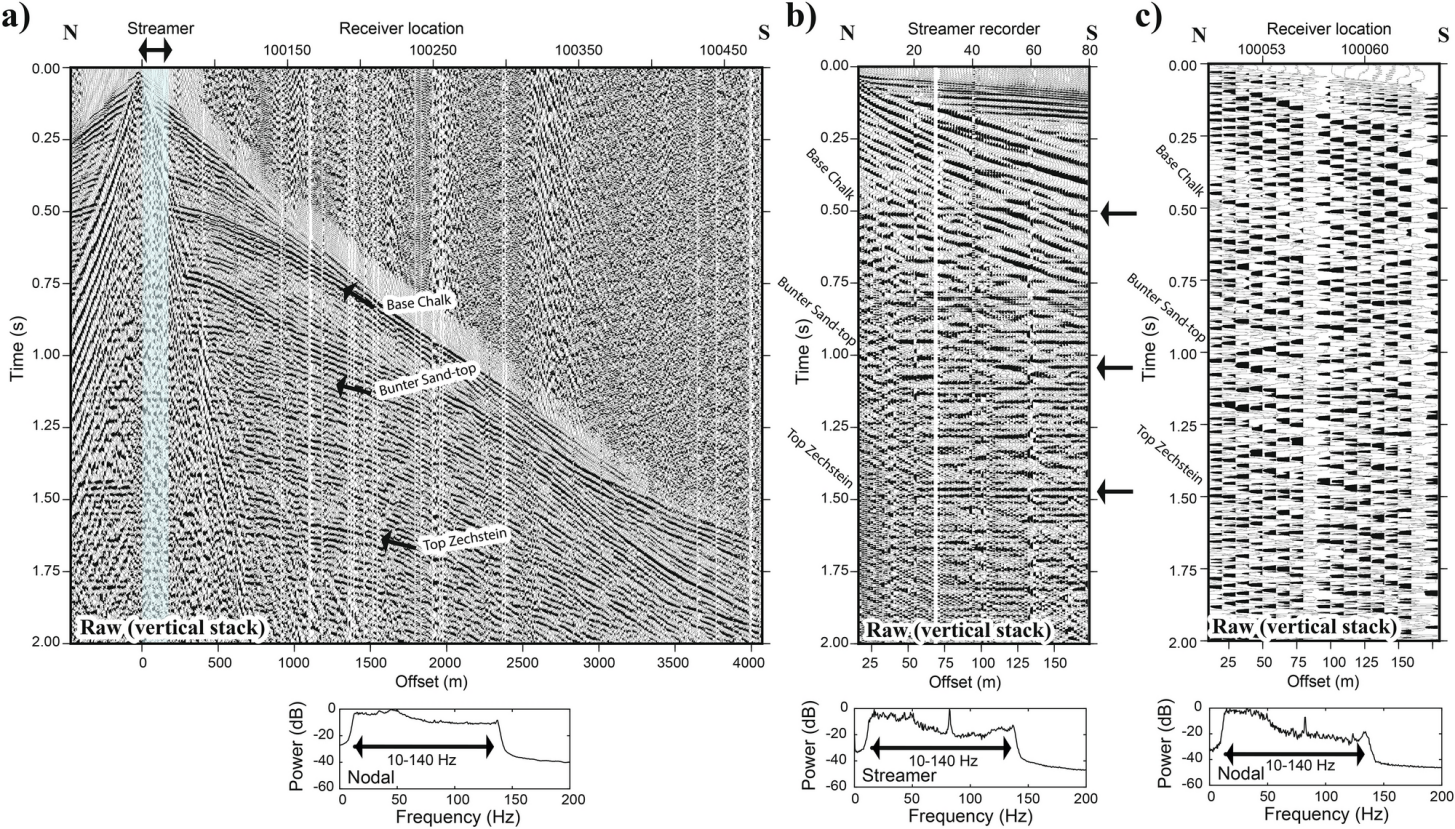
(**a**) An example of a raw gather from P10 for nodal recorders and corresponding amplitude spectrum, and (**b**) identical data recorded on the landstreamer sensors. The blue region in (**a**) mark the location of the landstreamer data. (**c**) A zoomed-in portion of the nodal recorders with equivalent offset and position of the landstreamer data. Note at near offsets, the landstreamer data better sample the entire seismic wavefield and certainly show better bandwidth compared to the nodal recorders since they are broadband but also better spatially sample high frequencies given their shorter spacing on the streamer. Generic Mapping Tools (GMT) V4.5 (https://www.soest.hawaii.edu/gmt/) was partly used to produce the figure for this study.

Figure 4a shows an example of a raw shot gather (after cross-correlation and vertically stacking of the three repeated shot records) for the active nodal array of approximately 4.5 km length along a portion of P10. The corresponding raw data from the landstreamer recorders are shown in Fig. 4b and a pulled-out portion for equivalent offsets from the nodal recorders are shown in Fig. 4c with their amplitude spectra, respectively. Overall, data quality is good for both datasets given this was acquired along a busy road. Nevertheless, when comparable portions are carefully looked at (i.e., Fig. 4b,c), the landstreamer data show a series of reflections with higher quality and strength down to 1.5 s than the nodal data for identical offsets of both datasets. The recorded bandwidth is also broader in the landstreamer than the nodal recorders. A cyclic signal at 82 Hz is seen strongly in the near offset data on both datasets, which we interpret as an amplified second harmonic of 41 Hz likely generated from the cooling/heating fan systems of the seismic vibrators. The national grid 50 Hz fundamental frequency is only seen in the nodal data since geophones are coil-based and affected than the landstreamer recorders. This is a farther advantage of digital recording for onshore applications since they are less affected by various cultural noises^39^.

**Fig. 5:**
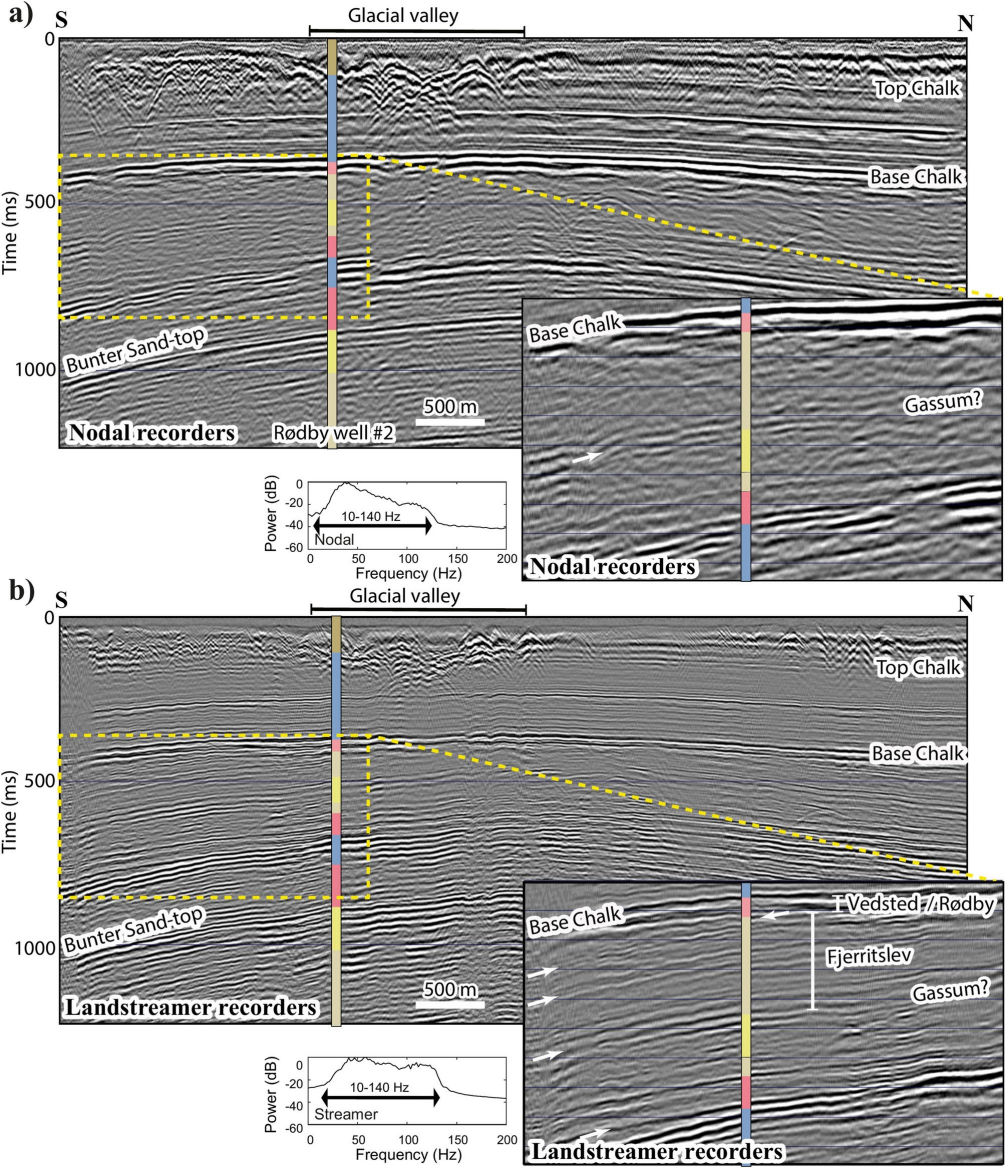
(**a**) Unmigrated brute stacks of the southern portion of P10 for (**a**) nodal (with a nominal CDP fold of 350) and (**b**) landstreamer (with a nominal CDP fold of 40) recorders. Identical stratigraphic column as shown in Fig. 1 is presented here (for the outset the stratigraphic column is offset to the middle of the sections) to facilitate horizon tracking. A close-up of the section and its corresponding amplitude spectrum for these two different recording elements are also shown to allow better comparison between the two datasets. Note the higher resolution and broader bandwidth of the landstreamer data despite shorter offsets and fold coverage for the top 1000 ms. A thin layer parallel to and near below the base of the Chalk Group toplapped (unconformable truncated) with a series of outward underlying dipping reflections is interpreted to represent the Vedsted/Rødby seal formations. White arrows show a series of toplap or truncation (for Fjerritslev) and onlap (for Oddesund) reflections in the landstreamer data, which are hard to identify in the nodal data. These features are characteristics for erosional surfaces (periods), where depositional environment changes. Generic Mapping Tools (GMT) V4.5 (https://www.soest.hawaii.edu/gmt/) was partly used to produce the figure for this study.

Figure 5 shows an example of unmigrated brute stacked section of P10 jointly processed together with P11 using a 5 m CDP spacing for nodal (> 350 CDP fold) and landstreamer (< 40 CDP fold) data. The streamer data show superior resolution and image internal structures e.g., toplap reflections truncated near the base of the Chalk Group as compared with the nodal data. The interpreted Gassum Formation is also better seen in the landstreamer data. More importantly a thin layer, interpreted to be the Vedsted and Rødby formations, is better imaged by the landstreamer data. Other features such as several diffraction signals, a glacial channel near the surface are imaged by both datasets. The landstreamer data show reflectivity down to the target Bunter Formation Sandstone at about 1000 ms, which is promising and trustworthy. Nonetheless, we do not want to argue to replace the nodal recorders, rather to illustrate that the broadband and fine spatial sampling provided by this dual-element acquisition setup allows complementary high-resolution data. It helps to unravel important internal structures for CCS applications. The better spacing sampling of the streamer recorders (and shorter offsets) helps to capture the generated sweep frequencies and show minimum aliasing issues for slower arrivals e.g., shear- and surface-waves. Given the moveout insensitivity of the landstreamer data (maximum offset of 200 m) at later arrivals, the nodal recorders with much greater offsets (Fig. 4) help to constrain the velocities and provide deeper imaging capabilities. Therefore, both datasets combined results in improved resolution and imaging at full depth-scale range like that of the marine seismic solution.

## De-risking CCS using the dual-element seismic recording

**Fig. 6:**
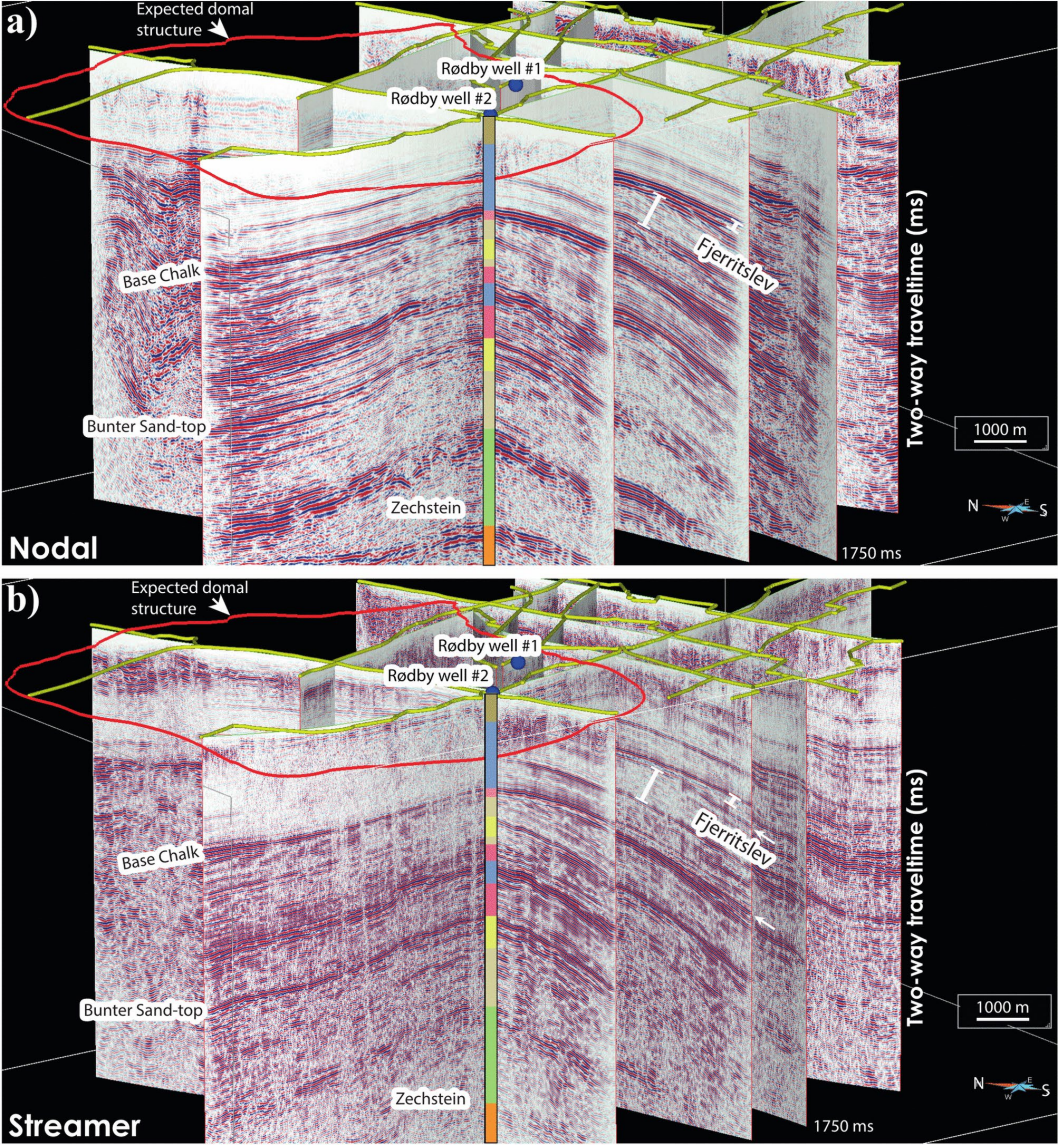
3D visualization of the migrated stacked sections of (**a**) nodal and (**b**) landstreamer data (shown in time for 1750 ms), separately processed to show their complementary nature for CCS applications. While complementary, several key features, as marked by the white arrows, are better imaged at shallow traveltimes by the landstreamer data while the nodal recorders typically show better imaging capabilities at depth. In particular, toplap and onlap reflectivity from Fjerritslev and Oddesund formations, respectively are better seen in the landstreamer data. SKUA-GOCAD 17 software (https://www.aspentech.com/en/products/sse/aspen-skua) is used for 3D visualization and to prepare the figure for this study.

Figure 6 shows 3D visualization of all the seismic profiles (migrated in time) for both nodal and landstreamer arrays. While the nodal arrays have higher CDP fold and better image deeper structures, the overall features are well imaged in both datasets. As expected along most profiles, the near surface is better imaged by the landstreamer data while the main C0_2_ target reservoir, Bunter Sand Formation and Zechstein Group are better imaged in the nodal data. A careful and thorough inspection of the data, however, provides interesting observations and how the landstreamer recorders can help de-risking CCS development at the Rødby site.

**Fig. 7:**
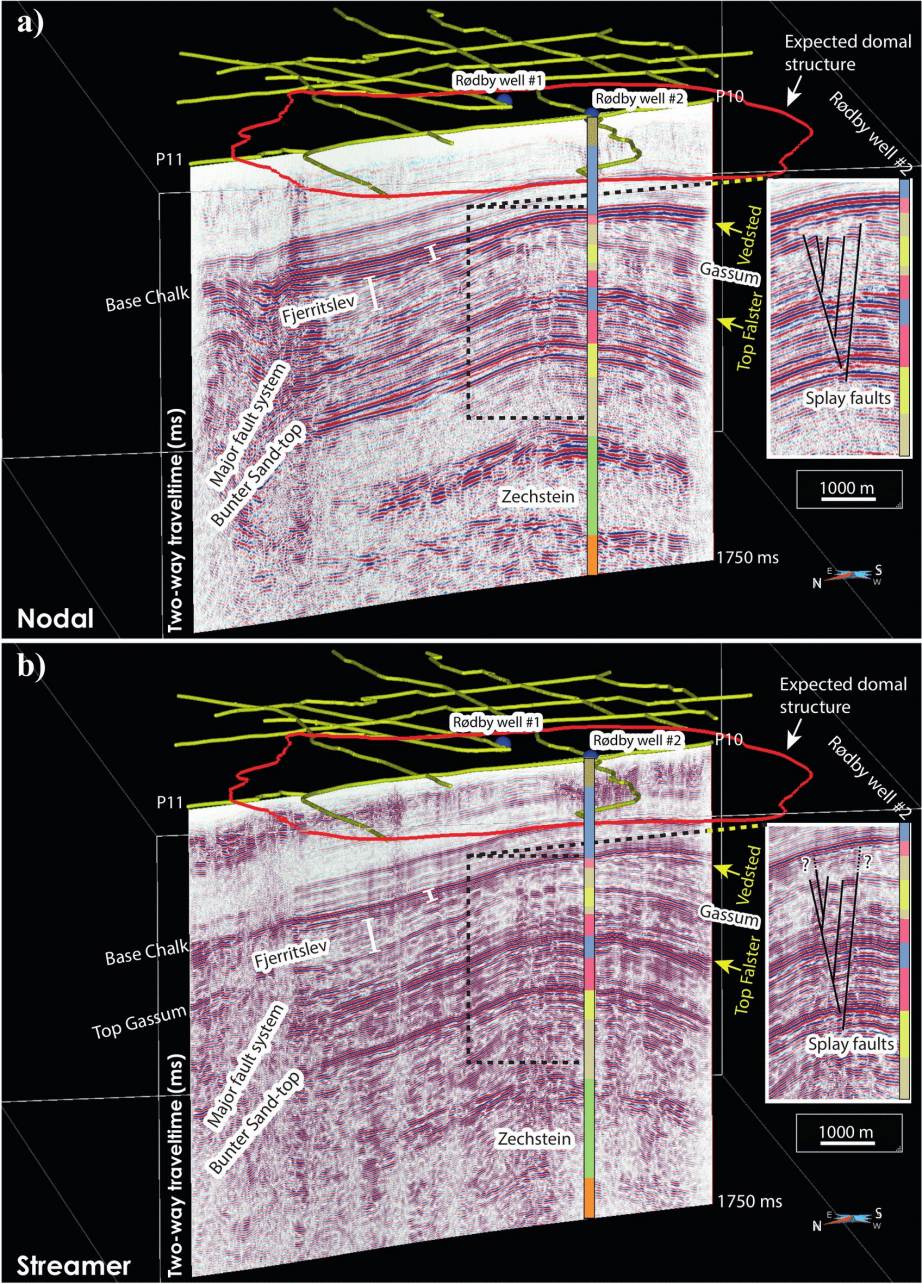
Migrated stacked section of (**a**) nodal and (**b**) landstreamer data (presented in time for 1750 ms), separately processed but jointly for P10 and P11. A close-up of each section showing a small graben-type structure is presented as inset figure with the stratigraphic column as observed in the Rødby-2 well. A key observation here is the thin layer between the base of the Chalk Group and toplap features of the Fjerritslev Formation that we interpret to be the late Cretaceous Vedsted and Rødby formations, which would be collectively a secondary seal for the Bunter Sandstone Formation reservoir. The landstreamer data suggest this shallow quaterly seal is slightly displaced hence the splay faults have penetrated through this final seal. Top Gassum is also better imaged in the landstreamer data while deeper formations such as Bunter and Zechstein are better imaged in the nodal data. SKUA-GOCAD 17 software (https://www.aspentech.com/en/products/sse/aspen-skua) is used for 3D visualization and to prepare the figure for this study.

Figure 7 demonstrates the most striking features of the landstreamer data. Not only the landstreamer data better resolve the toplap or reflection truncations of the Fjerritslev Formation (truncated below the base of the Chalk Group), but they also show a thin layer of Vedsted and Rødby formations, underlying the base of the Chalk Group. Vedsted and Rødby formations have an opposite polarity with respect to the base of the Chalk Group (due to negative reflection coefficient) and this makes this recognition easier. Vedsted and Rødby formations, although thin, are shallow seals for the Bunter Sandstone Formation reservoir, hence their recognition is important to ensure an additional level of integrity for potential CO_2_ injection at the Rødby site. This reflectivity is also partly imaged in the nodal data but with much smoother character and less resolution.

The top of the Gassum Formation is imaged in both datasets. Nevertheless, the landstreamer data better show the Top Gassum as a single reflection underlying the Fjerritslev Formation especially on the northern flank of the domal structure (Fig. 7). On the northern portion the seismic section (P10 and P11 are jointly processed; see Fig. 1), a major fault cuts through the entire stratigraphy as evident from the disturbed and discontinuous reflectivity. This is a regional fault striking east–west of the area and occurs on the most northern flank of the Rødby structure dome^36^. Its presence does not jeopardize CO_2_ injection since it occurs on the lower flank of the dome. Within this major fault system, the Gassum Formation is better imaged in the landstreamer data allowing to better track this reservoir than in the nodal data. This is an important observation if Gassum Formation is for example used for thermal storage applications at the Rødby site and if a conflict between the two purposes (thermal versus CO_2_ storages) emerges.

In addition, the salt movement and withdrawal has created a graben-type structure with smaller faults offset the overlying stratigraphy originating from the top of the Zechstein Group all the way through the Gassum Formation. They form a series of splay faults (Fig. 7) with normal faulting mechanism. While there is no doubt that the overlying primary and secondary seals (Ørslev and Falster formations) are faulted, the Top Gassum Formation is evidently also downfaulted, displaced, into different blocks and that the landstreamer data show this slightly better. A question here remains if the Vedsted and Rødby formations, to be an ultimate seal, are also faulted. The base of the Chalk Group appears undisturbed in both datasets on the crest of the dome, a crucial place for CO_2_ plume emplacement and potential leakage.

The Fjerritslev Formation is considered as an additional secondary seal for Bunter Sandstone Formation in case CO_2_ leaks from Ørslev and Falster formations through permeable fault networks. The toplap or truncated Lower Jurassic Fjerritslev Formation reflections are evidently better imaged in the landstreamer data and in most places also the overlying Lower Cretaceous Vedsted and Rødby formations, which are separated by an unconformity surface. Lowermost Cretaceous and the entire Middle and Upper Jurassic successions are eroded away at the Mid-Cimmerian unconformity as imaged near Top Fjerritslev with clear truncations overlain by Lower Cretaceous, indicating uplift and erosion of the Rødby structure. These toplaps structures have not been intersected by any boreholes, or at least recognized, in the area and is for the first time observed showing the potential of the landstreamer system for mapping detailed internal structures and depositional and erosional environment on the flanks of the domal structures. Should CO_2_ leak from the splay faults, these internal layering and structures including their orientations may influence migration of CO_2_ plume at higher stratigraphic levels.

Another important feature of the datasets is the glacial valley (coarse-grained glacial channel and sediments) imaged in higher resolution carving through the Post Chalk Group in the landstreamer data (Figs. 5 and 7). These glacial sediments are the source of groundwater in most part of Denmark for both drinking and farming. It is important that they are imaged and well characterized to mitigate that any CO_2_ leakage may find its way into these deposits through the splay faults and becomes an environmental problem and unsafe for CO_2_ storage.

Finally, the Rødby seismic data were used to estimate potential CO_2_ capacity through a simulation work and assuming a range of porosities. Based on a deepest closure of the Bunter Sandstone Formation estimated to be around 1415 m and its top around 1100 m, approximately 300 m relief and an area of 115 km^2^ is present for CO_2_ injection at Rødby^36^. The Rødby structure is currently a target for further exploration of potential CO_2_ sequestration, and more work and data will emerge to support its development work. 3D seismic data in particular should be acquired since true geometry and character of the reflections can only reliably be obtained using 3D methods. The current survey, however, justifies why such a 3D dataset should be acquired given the potential of the site for GCS purposes. The dual-element survey and setup have played an important role for its initial characterization and site selection. Based on the results, we recommend more technical development work and solutions but also trials for onshore GCS site characterizations. They are certainly worth to try, as the need and push for the net-zero solutions accelerates, and courtiers especially in Europe show a thirst for successful mitigation of CO_2_ emission.

## Conclusions

The Rødby site has allowed for the first time to illustrate new detailed information from the landstreamer recorders for de-risking CCS applications. In a dual-element recording system, both nodal and landstreamer data provide complementary information for a full-depth scale imaging of the stratigraphy and key structures important for the integrity of CO_2_ storage and its migration. Given the finer sampling spacing of the landstreamer recorders, but also here their broadband nature, it was possible to retain the sweep frequencies generated by the seismic vibrators. This broadband nature and improved spatial sampling at near offsets helped the landstreamer data to image in high and improved resolution the thin sequence of the secondary seals belonging to the late cretaceous Rødby and Vedsted formations. While the landstreamer array can be used alone, we recommend to complement this with nodal arrays since logistical challenges and road accessibility may hinder sufficient signal quality for deep imaging. Long offsets in nodal arrays (in this study a maximum of 7500 m was used) also help obtaining velocity information at depth, which is not possible with only landstreamer arrays. A small graben structure is clearly imaged to originate from the top of the Zechstein Group due to structural growth and doming offsetting the entire stratigraphy including the Ørslev and Falster seal formations. The landstreamer data image the target reservoir; The Bunter Sandstone Formation is imaged downfaulted due to salt withdrawal so the overlying Gassum Formation. The Gassum Formation is faulted by a series of splay faults, and it is possible to see its reflection and fault blocks better in the landstreamer data. The landstreamer data show clear toplap features from the Lower Jurassic Fjerritslev Formation, which are truncated and overlain by the Lower Cretaceous Rødby and Vedsted formations. This truncation thus demonstrates a major hiatus possibly related to significant regional uplift and erosion due to elevation of the Rødby domal structure. One or two of the splay faults appears to penetrate through the uppermost seals of Rødby and Vedsted formations, which is clearly shown in the landstreamer data compared to the nodal data. The tailored recoding system, therefore, shows its capability as a cost and time effective recording tool for CCS applications onshore and is a step forward for site characterization and detailed stratigraphic imaging. It also helps to provide a full-scale imaging possibility in case conflicts for the subsurface water and energy extraction, energy and CO_2_ storage usages become an issue.

## Methods

### Seismic data acquisition and processing

The seismic data acquisition at the Rødby site included 11 profiles (Fig. 1) summing to approximately 110 km of 2D reflection seismic surveying. Acquisition began on 11th June 2023 and completed on 27th July 2024. An additional of two days were allocated for data harvesting and packing of the equipment. Daily operation consisted of several teams rotating in shifts of in total 8–10 people. The plan for the daily progression was set ahead, and work zones along the roads were marked by road signs for safe operation. The lengths of working zones were on average 2.5 km a day. Within the operational zone, two 12t seismic vibrators were utilized to generate synchronized seismic sweeps every 10 m along the profiles. The sweep parameters were designed to be linear with bandwidth 10–140 Hz and length of 18 s, based on the previous surveys (e.g., Stenlille site^31^). Prior to accepting these sweeps, a series of tests were conducted to ensure that the sweep frequencies and length provided quality data at Rødby. After this initial test and data quality checking ensuring also that the two seismic sources were synched in time and phase, the acquisition was permitted. Temperature changed significantly during the survey as well as ground conditions and these led to a few of instances (not more than a half a day in total) where only one vibrator functioned until the second one returned from services. The acquisition setup consisted of acquiring simultaneously data along the 2 m-spaced MEMs-based land-streamer, and a 10 m-spaced nodal array (connected to 10 Hz geophones). Shots were generated at the nodal receivers i.e., 10 m spacing. The sampling rate of the nodal recorders was set to 2 ms, while for the MEMS units sampling was 1 ms. This sampling was justified given the sweep range of 10–140 Hz used for the seismic survey. A total listening time of 25 s was used to ensure at least 5 s data can be used for data processing and for imaging purposes. Considering that landstreamer units were dragged along with the seismic vibrators, the coordinates of the units were interpolated based on the surveyed coordinates from the fixed nodal recorders.

Each seismic profile was independently processed given the high-quality nature of the landstreamer data as presented in the raw shot gathers and brute stacks in Figs. 4 and 5; however, the overall processing flow was kept consistent, with only some variations. Supplementary Table S1 lists the key processing steps used for both datasets. The 25 s uncorrelated seismic records were cross-correlated with the 18 s theoretical 10/140 Hz linear sweep, and 5 s corelated seismic records were used for the data processing. Assuming an average subsurface seismic velocity of 3000 m/s, this would permit a maximum imaging depth of 7–8 km. After correlation, shot gathers were inspected for abnormalities, such as noisy traces to be discarded. The traces were binned into common midpoint (CMP or CDP here) bins, each centrally located along a smooth spline curve that traversed the points of highest CMP density based on the nodal sensor locations. The CDP spacing was set to 5 m for the nodal data and similarly for the streamer data to allow direct comparison and merging if needed. For consistency, the same crooked processing line was used to bin the streamer data.

Effects of different processing steps are illustrated for the shot gather presented in Fig. 4 in Supplementary Figs. S2 and S3. Processed data undergone a number of key steps and in particular after bandpass filtering, refraction static corrections and a tailored surface-wave denoising, the quality of the reflections witnessed remarkable improved quality higher and it was possible to track marker horizons such as Top Zechstein and Base of the Chalk Group; this is more evident in the landstreamer data (Fig. S3). A tailored non-aggressive surface-wave attenuation filter was designed on a cone where the surface-waves were judged to be present and then a curvelet-based attenuation filter was designed to only operate within this cone in order to attenuate the surface-waves. This helped to attenuate anomalous surface-wave amplitudes without touching the reflection signals at early traveltimes.

### Horizon reflection statics

Unlike the other datasets acquired for CCS site characterization onshore Denmark, we decided not to merge the two datasets for the Rødby area, because the landstreamer data revealed already at the brute stack stage detailed information that we did not want to lose at the expense of merging the two datasets. Given the less sensitivity of the landstreamer data to normal moveout corrections (NMO), a new approach, however, had to be developed to correct for any remaining static issues on the landstreamer data using the nodal sections. This was the only time that the nodal data were used to support processing the landstreamer data. Supplementary Figure S4 shows an example of this workflow for merged P10 and P11 profiles. The Top Chalk appears very continuous in both final processed landstreamer and nodal sections. To ensure the same level static corrections for both datasets, this horizon was picked in the unmigrated stacked sections of both datasets and the difference in time was added to the landstreamer picks to account for any mismatch, static issues, for the continuity of the reflection. While the difference was not significant, it helped to improve the overall continuity of the reflections in the landstreamer data.

### Decimating landstreamer and nodal array data

To better judge if the landstreamer recorders have better quality given their broadband nature, we decimated this data set to that of the nodal array recorders (i.e., every 10 m recorders were kept). We also decimated the nodal array dataset to contain only one side offset data and for a maximum of 180 m, corresponding to that of the landstreamer array. Supplementary Fig. S5 shows the resulting unmigrated stacked sections of these datasets. It is clear that the landstreamer data contain much higher quality and resolution in the section than the nodal array data. Faults are better observed including offsets in the reflections but also smaller features such as diffraction signals associated with faults.

## Supplementary Information

Supplementary Information.

## Data Availability

All data and associated processing and interpretation report from the Rødby survey can be freely (open access) accessible from: https://www.geus.dk/produkter-ydelser-og-faciliteter/data-og-kort/ccs-data-2022–2024https://data.geus.dk/pure-pdf/GEUS-R_2024-18_web.pdf and https://pub.geus.dk/en/publications/ccs2022-2024-wp1-the-r%C3%B8dby-structure-seismic-data-and-interpretat.
