# Supplementary material for: Geological carbon storage site characterization using a dual element seismic recording technology
Source: Sci Rep. 2025 Apr 15;15:12937. doi: 10.1038/s41598-025-96012-8 (PMC12000295; doi:10.1038/s41598-025-96012-8)
Supplement: Supplementary file 1 — Supplementary Information. [file 41598_2025_96012_MOESM1_ESM.docx]

**Supplementary Information**

***Table S1****: Processing steps of both the landstreamer and nodal data of the Rødby dataset.*

|  | **Processing step** |  | **Details** |  |
| --- | --- | --- | --- | --- |
|  |  |  | *Landstreamer* | *Nodal recorders* |
| 1. | Import SEGD data |  | ✓ | ✓ |
| 2. | Cross-correlation with theoretical sweep |  | ✓ | ✓ |
| 3. | Vertical stack of repeat shots (diversity) |  | ✓ | ✓ |
| 4. | Spherical divergence |  | 1D velocity | 1D velocity |
| 5. | Conversion to minimum phase |  | ✓ | ✓ |
| 6. | Geometry setup and CDP binning |  | 5 m CDP spacing | 5 m CDP spacing |
| 7. | First arrival picking and computation of refraction statics |  |  | ✓ |
| 8. | Muting of noisy traces |  | ✓ | ✓ |
| 9. | Elevation statics |  | Topographic statics using replacement velocity of 2300 m/s and reference elevation of 15 m above sea level | |
| 10. | Bandpass filter |  | 10-30-130-140 Hz | 10-35-130-140 Hz |
| 11. | Airwave attenuation (330 m/s) |  | ✓ | ✓ |
| 12. | Sloped median filter |  | 500 m/s | 1000 m/s |
|  |  |  | 800 m/s | 2000 m/s |
| 13. | Refraction static corrections |  |  | ✓ |
| 14. | Constant velocity analysis |  | ✓ | ✓ |
| 15. | Reflection-based residual statics |  | One round | Two rounds |
| 16. | Gapped deconvolution |  | Gap length: 18-17 ms; operator length: … | Gap length: 18-17ms; operator length: … |
| 16. | First break top mute |  | ✓ | ✓ |
| 17. | NMO corrections |  | 50 % stretch mute | 50 % stretch mute |
| 18. | Stack |  | ✓ | ✓ |
| 20. | Bandpass filter |  | 20-40-110-130 Hz | 10-30-120-140 Hz |
| 21. | F-X deconvolution coherency filter |  | ✓ | ✓ |
| 22. | Amplitude balance |  | ✓ | ✓ |
| 23. | Finite-difference migration |  | ✓ | ✓ |
|  |  |  |  |  |


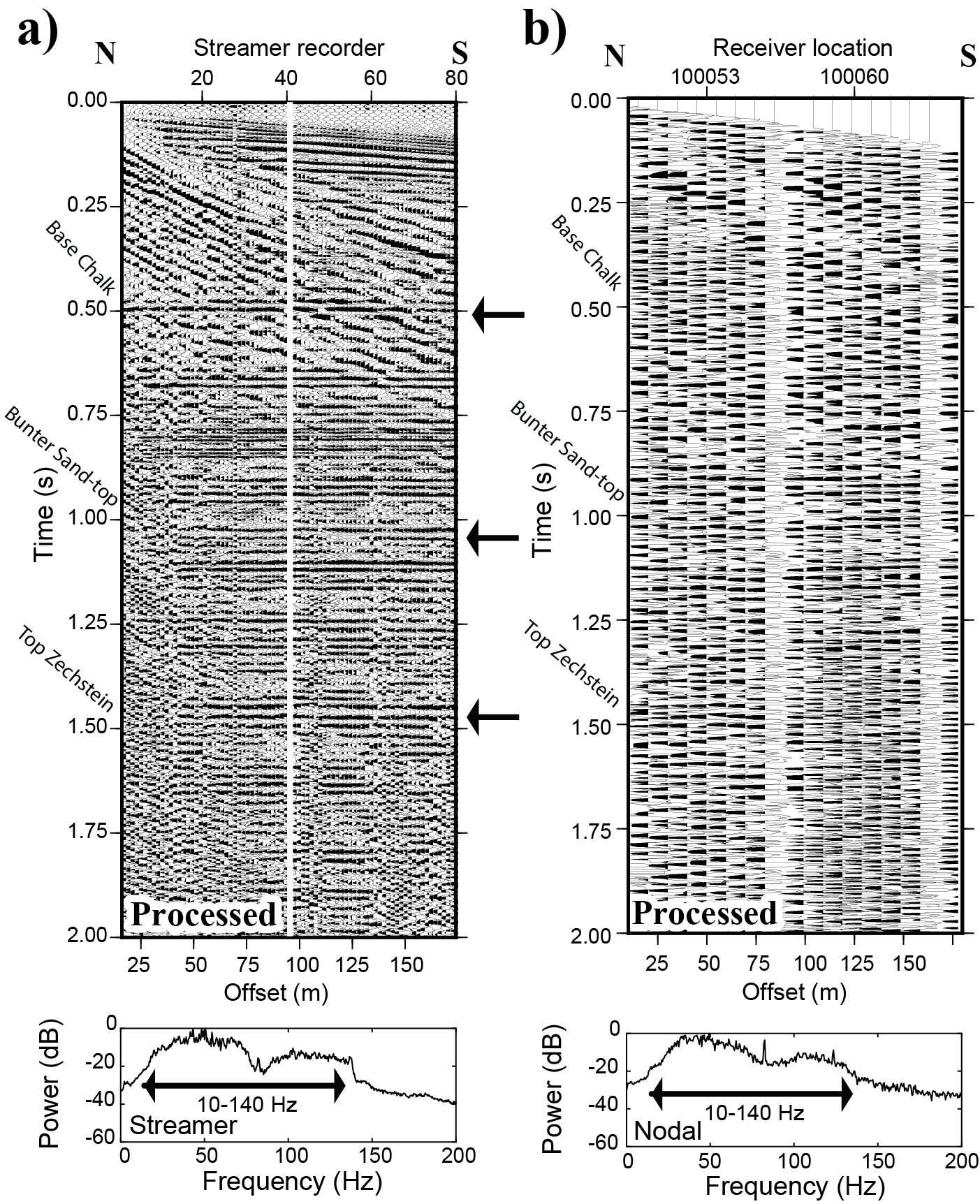


***Figure S1****: The same shot gathers as shown in* ***Figure 4b,c*** *in the main body (from P10) but fully processed in the shot gather domain for (a) the landstreamer (80 units and only vertical components) and (b) the wireless nodal recorders with their corresponding amplitude spectra shown below each panel. An AGC of 300 ms is applied for display.*

***
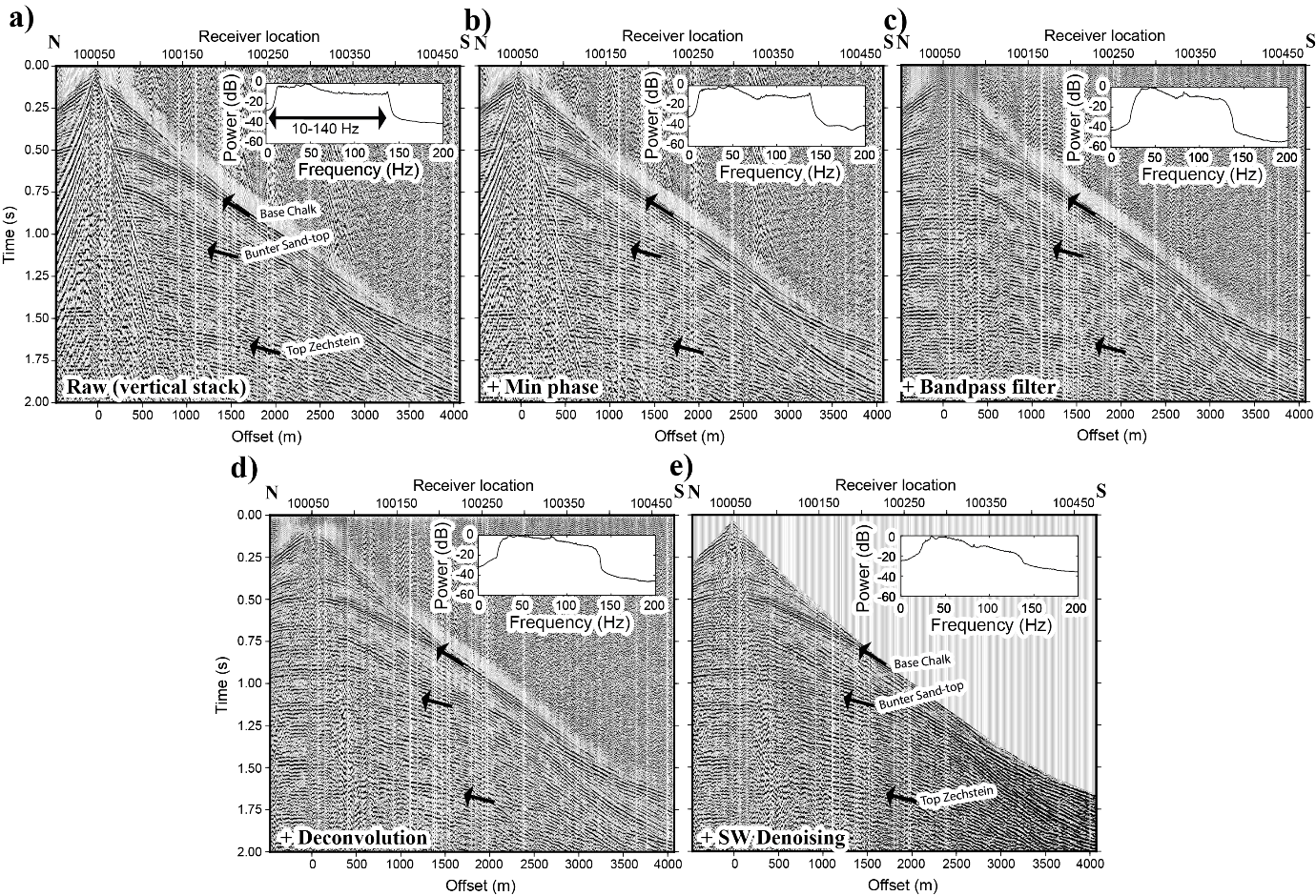
***

***Figure S2****: The same gather for nodal data as shown in* ***Figure 4a*** *in the main body (a) for the entire offset range, from P10, illustrating the quality of the data and improvement in signal-to-noise ratio at different stage of prestack processing workflow such as (b) minimum phase conversion, (c) bandpass filtering, (d) deconvolution and (e) a tailored surface-wave denoising work. The three key horizons, Base Chalk Group, Bunter Sandstone Formation and Top Zechstein Formation are labelled for facilitating the data quality judgment and improvements obtained.*

***
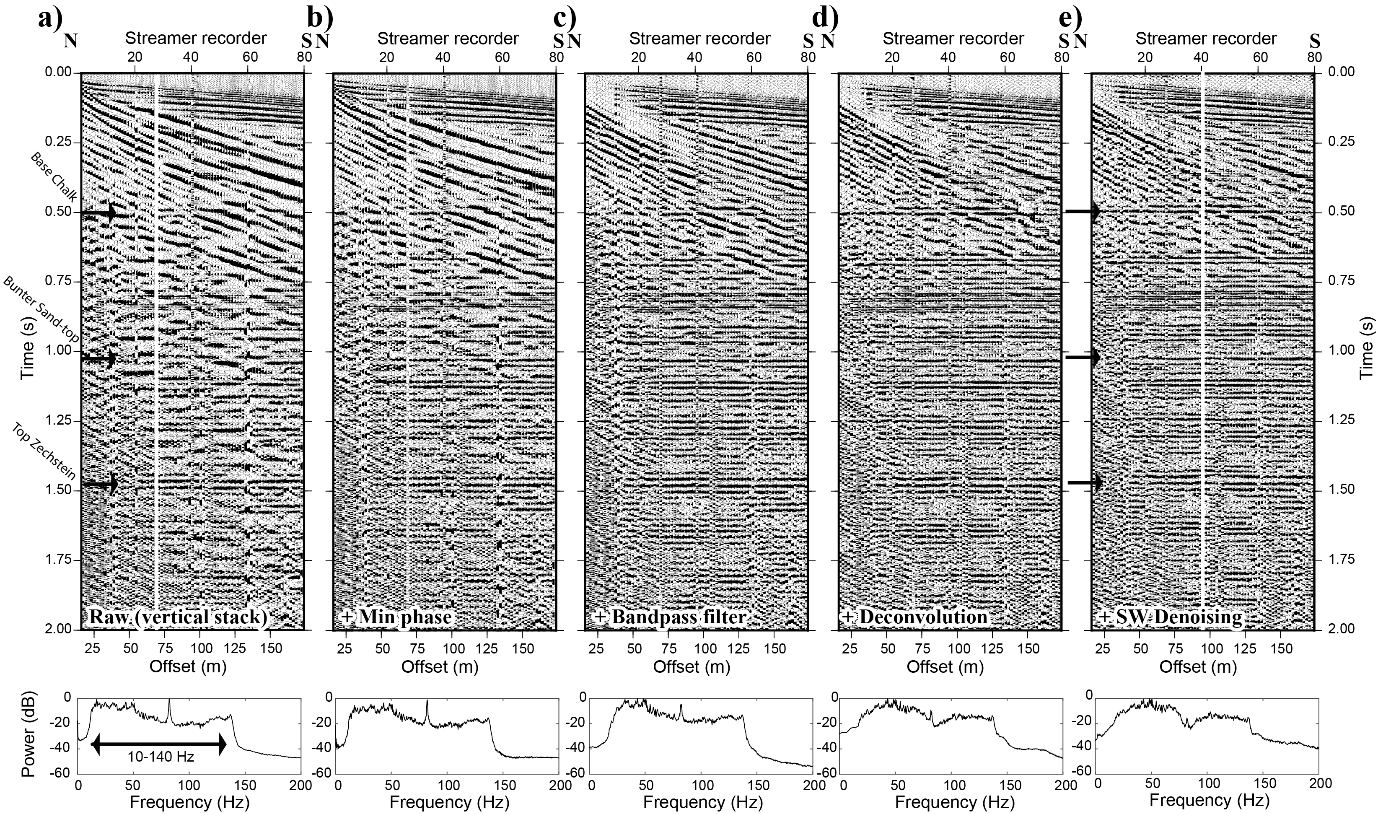
***

***Figure S3****: The same gather for landstreamer data as shown in* ***Figure 4b*** *in the main body (a) for the entire offset range, from P10, illustrating the quality of the data and improvement in signal-to-noise ratio at different stage of prestack processing workflow such as (b) minimum phase conversion, (c) bandpass filtering, (d) deconvolution and (e) a tailored surface-wave denoising work. The three key horizons, Base Chalk Group, Bunter Sandstone Formation and Top Zechstein Formation are labelled for facilitating the data quality judgment and improvements obtained.*


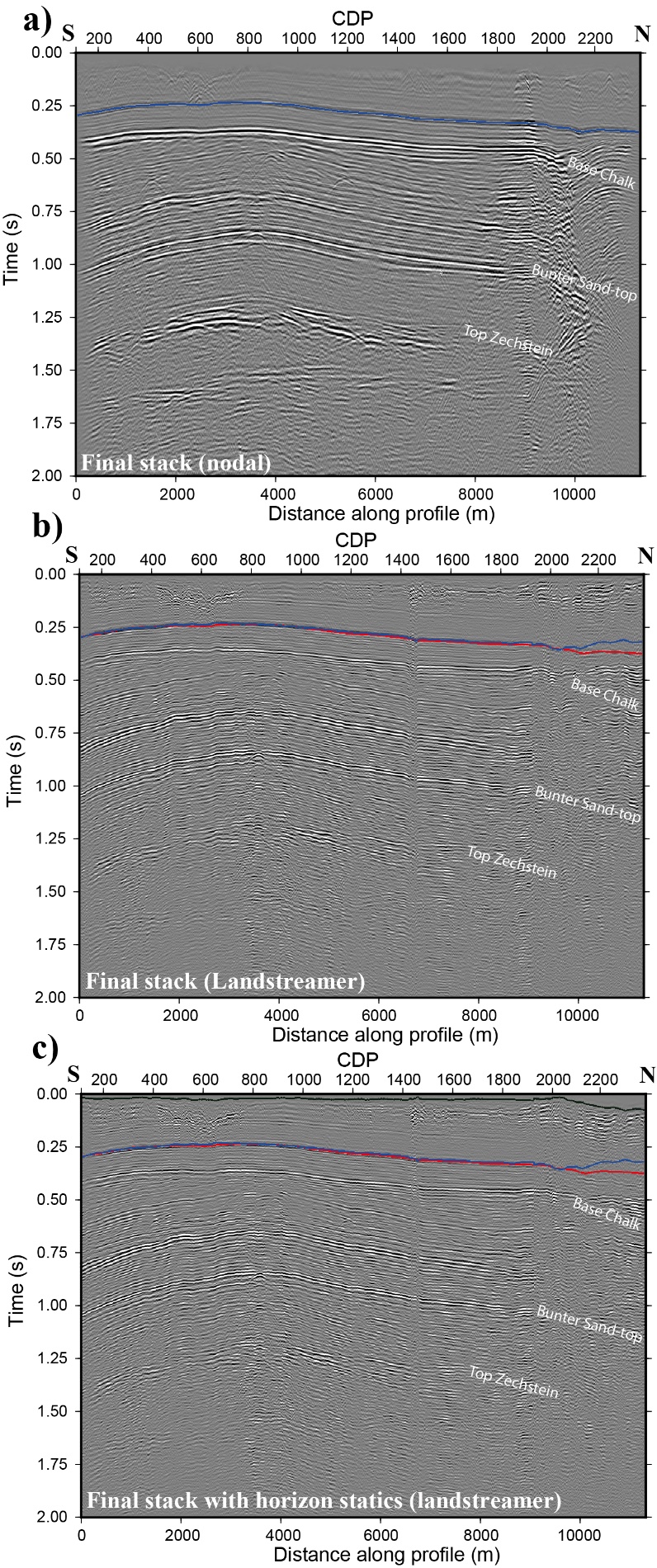


***Figure S4****: (a) The implementation of horizon statics (usually from the base of the Chalk Group) benefiting from the continuity of the nodal data. (b) Horizon picked (blue) to adjust the landstreamer data, and (c) horizon picked on the landstreamer data (blue) and the difference shown as black applied to the landstreamer final unmigrated stacked section (from P10). Judging the continuity and consistency, we think this approach was partly successful to solve the low fold and the static issues.*

***
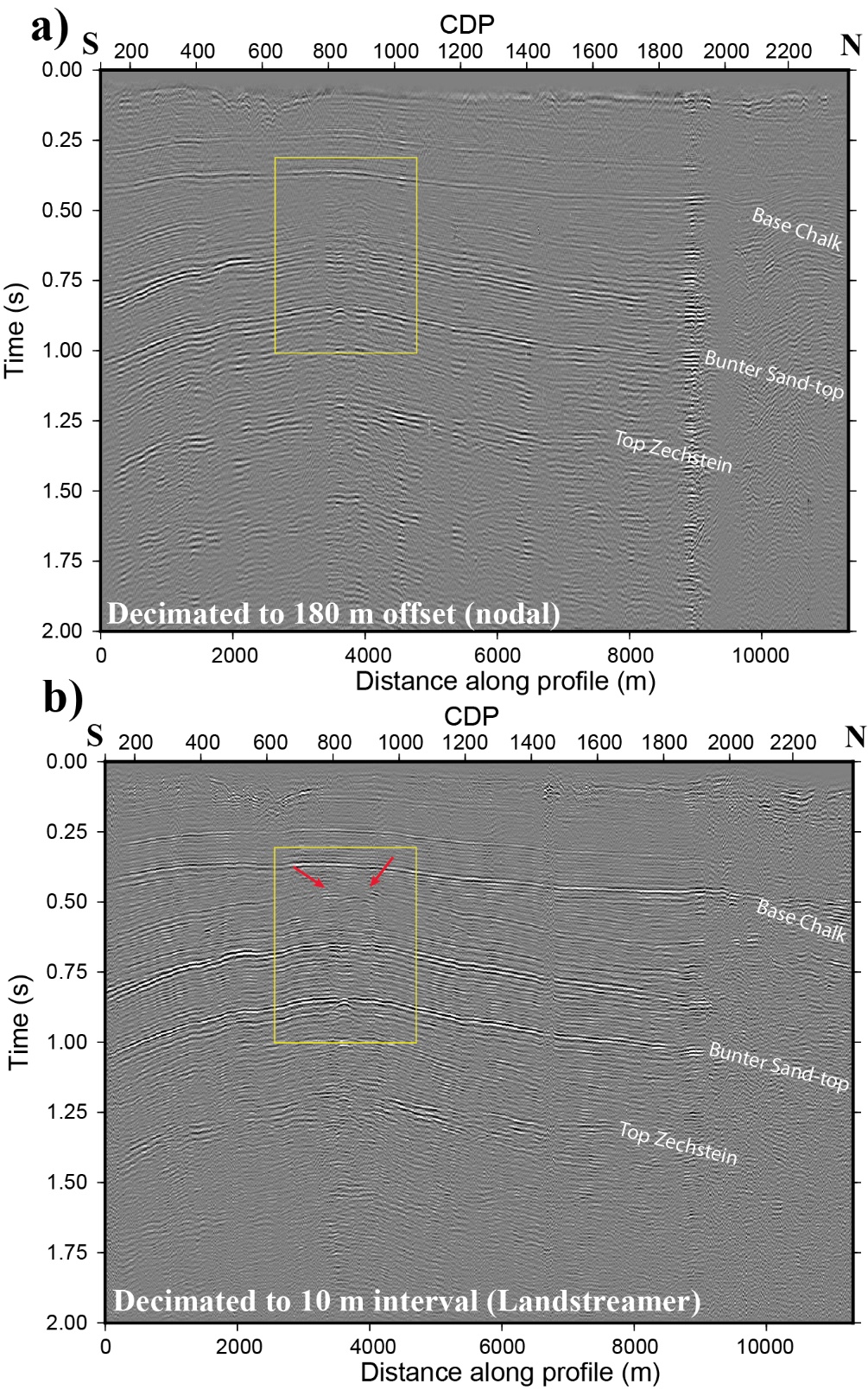
***

***Figure S5****: Comparison between unmigrated stacked sections of (a) nodal versus (b) landstreamer arrays, decimated to correspond to the same offset (kept up to 180 m for the nodal array data) and recorder spacing (kept 10 m for the landstreamer array data) between the two arrays. Higher quality and resolution data are evident in the landstreamer data specially within the yellow box where faults offset the Gassum Formation; note also minor features such as diffraction signals in the landstreamer data.*
